# Risk prediction for sporadic Alzheimer's disease using genetic risk score in the Han Chinese population

**DOI:** 10.18632/oncotarget.6271

**Published:** 2015-11-02

**Authors:** Qianyi Xiao, Zhi-Jun Liu, Sha Tao, Yi-Min Sun, Deke Jiang, Hong-Lei Li, Haitao Chen, Xu Liu, Brittany Lapin, Chi-Hsiung Wang, S. Lilly Zheng, Jianfeng Xu, Zhi-Ying Wu

**Affiliations:** ^1^ Center for Genomic Translational Medicine and Prevention, School of Public Health, Fudan University, Shanghai, China; ^2^ Department of Neurology and Research Center of Neurology in Second Affiliated Hospital, and the Collaborative Innovation Center for Brain Science, Zhejiang University School of Medicine, Hangzhou, China; ^3^ Department of Neurology and Institute of Neurology, Huashan Hospital, Fudan University, Shanghai, China; ^4^ State Key Laboratory of Genetic Engineering and Ministry of Education Key Laboratory of Contemporary Anthropology, School of Life Sciences, Fudan University, Shanghai, China; ^5^ Program for Personalized Cancer Care, NorthShore University Health System, Evanston, IL, USA; ^6^ Fudan Institute of Urology, Huashan Hospital, Fudan University, Shanghai, China

**Keywords:** Alzheimer's disease, genetic risk score, risk prediction, single nucleotide polymorphism, association, Gerotarget

## Abstract

More than 30 independent single-nucleotide polymorphisms (SNPs) have been associated with Alzheimer's disease (AD) risk by genome-wide association studies (GWAS) in European. We aimed to confirm these SNPs in Chinese Han and investigate the utility of these genetic markers. We randomly divided 459 sporadic AD (SAD) patients and 751 cognitively normal controls into two sets (discovery and testing). Thirty-three SAD risk-associated SNPs were firstly tested in the discovery set. Significant SNPs were used to calculate genetic risk score (GRS) in the testing set. Predictive performance of GRS was evaluated using the area under the receiver operating characteristic curve (AUC). In the discovery set, 6 SNPs were confirmed (*P* = 7.87 × 10^−11^~0.048), including rs9349407 in *CD2AP*, rs11218343 in *SORL1*, rs17125944 in *FERMT2*, rs6859 in *PVRL2*, rs157580 and rs2075650 in *TOMM40*. The first three SNPs were associated with SAD risk independent of *APOE* genotypes. GRS based on these three SNPs were significantly associated with SAD risk in the independent testing set (*P* = 0.002). The AUC for discriminating cases from controls was 0.58 for GRS, 0.60 for *APOE*, and 0.64 for GRS and *APOE*. Our data demonstrated that GRS based on AD risk-associated SNPs may supplement *APOE* for better assessing individual risk for AD in Chinese.

## INTRODUCTION

Alzheimer's disease (AD) is a neurodegenerative disorder characterized with progressive deterioration in cognition and behavior. AD is the most common form of dementia in aging population with a worldwide prevalence of 35.6 millions in 2010, and is expected to increase to 115.4 millions by 2050 [1]. In China, the burden of AD increased considerably in recent years due to aging population. The incidence of AD among people aged 60 years or older was 6.25 cases per 1000 person-years in 2010 in China [2]. Because several risk factors have been associated with AD risk [3], the disease may be preventable by reducing these risk factors. It is widely believed that targeted prevention for subjects with higher risk for AD is likely a more effective strategy.

AD is highly heritable and its heritability is estimated up to 76% [4]. Previous work suggested that genetic variants play an important role in the development of the disease. Mutations in *APP*, *PSEN1* and *PSEN2* lead to early onset familial AD [5-8]. *APOE ε4* allele has been found to be the strongest risk factor for sporadic AD (SAD), the most common form of AD including early- and late-onset SAD [9-12]. However, because about 40-50% of SAD do not carry the *APOE ε4* allele [11, 13], additional genetic variants that are related to SAD risk likely exist. Several genome-wide association studies (GWAS) in European descent have identified a number of independent AD risk-associated single nucleotide polymorphisms (SNPs) [14-19]. A recent meta-analysis combined four of these AD GWAS samples identified additional 11 independent AD susceptibility SNPs [20]. To date, 11 of these AD risk-associated SNPs (in 9 genes) have been reported to be significantly associated with AD risk in the Han Chinese population [21-27].

Although these AD risk-associated SNPs have a modest effect size individually (odds ratio [OR] of each individual risk allele is typically < 1.3), it is hypothesized that these SNPs may confer a stronger cumulative effect to AD. A genetic risk score (GRS) that captures the cumulative effect of SNPs has been comprehensively studied to stratify individual risk in several complex diseases [28-31]. In this study, we aimed to first determine which AD risk-associated SNPs reported in European descent are associated with SAD risk in Han Chinese in a discovery set, and then to calculate GRS using these implicated SNPs and asses its discriminative performance in a testing set.

## RESULTS

### Key demographic and clinical information of study subjects

After quality control analyses, 1210 subjects retained in the study, including 459 SAD patients and 751 control subjects. Key demographic and clinical information for these subjects is presented in Table 1. Because the study was frequency matched for sex, no statistically significant difference in proportion of gender was found (*P* > 0.05). However, due to the frequency match for age (within 5-years), the mean age at examination was slightly, but statistically significantly younger in cases (71.2 years) than controls (72.7 years), *P* = 0.004. The age at onset in cases ranged from 45 to 88 years, with a mean of 68.5 years. About 35% SAD cases were early age onset (< 65 years). The mean MMSE score was significantly lower in cases (14.7) than in controls (25.1), *P* < 0.001. Similarly, *APOE ε4* carrier rate was significantly higher in cases (41.4%) than in controls (20.0%), *P* < 0.001.

These subjects were randomly assigned to the discovery set (232 cases and 373 controls) and testing set (227 cases and 378 controls). As shown in Table S1, there was no significant difference between the case subjects from the discovery and testing sets (*P* > 0.05), except that mean age at onset was slightly older in the discovery set (69.4 years) than in the testing set (67.6 years), *P* = 0.048.

Among 33 SNPs selected in the study, 3 SNPs were excluded due to genotyping failure (rs10838725) and minor allele frequency (MAF) < 0.01 (rs7274581 and rs12989701). None of the SNPs significantly deviated from Hardy-Weinberg equilibrium (HWE) among control subjects at *P* < 0.001. In the following analysis, 30 SNPs were analyzed.

**Table 1:** Characteristics of study subjects in the entire cohort

Characteristic	SAD (n=459)	Controls (n=751)	*P* value
Age at examinationa, mean ± SD, yr	71.2±9.6	72.7 ± 5.9	0.004
Age at onset, mean ± SD, yr	68.5±9.7		
Age at onset <65, n (%)	161(35.1)		
Age at onset ≥65, n (%)	298(64.9)		
Sex^a^, n (%)			0.407
Male	228(49.7)	354(47.1)	
Female	231(50.3)	397(52.9)	
MMSE score, mean±SD	14.7±6.6	25.1±3.5	<0.001
Missing	1		
APOE, n (%)			<0.001
APOE ε4 carriers	190 (41.4)	150 (20.0)	
Non-APOE ε4 carriers	269 (58.6)	601 (80.0)	

Abbreviation: SAD, Sporadic Alzheimer's disease; MMSE, Mini-mental state examination; SD, Standard deviation.

a Frequency matched

### Association of SAD risk with candidate SNPs in the discovery set

In the discovery set, 6 of the 30 SNPs were significantly associated with SAD risk in Chinese (*P* < 0.05) after adjustment of sex and age (age at onset for SAD patients and age at examination for control subjects) (Table 2). These 6 SNPs were rs9349407 at 6p12 in *CD2AP* (*P* = 1.23 × 10^−4^), rs11218343 at 11q24 in *SORL1* (*P* = 8.32 × 10^−4^), rs17125944 at 14q22 in *FERMT2* (*P* = 0.048), rs6859 at 19q13 in *PVRL2* (*P* = 0.001), rs157580 at 19q13 in *TOMM40* (*P* = 0.036) and rs2075650 at 19q13 in *TOMM40* (*P* = 7.87 × 10^−11^). The direction of association was consistent with that of European descent for all 6 SNPs (Table 2). Four of these SNPs (rs9349407, rs11218343, rs17125944, and rs2075650) remained significant after adjusting for *APOE* genotype, *P* < 0.05. Meanwhile, we analyzed LD between SNPs at 19 chromosome and *APOE* genotype (treating *ε3* and *ε2* as the same allele and ε4 as another allele), and found the last SNP (rs2075650) was in strong LD with *APOE* genotype, *r*^2^ = 0.48 (Table S2). Associations between SNPs and SAD risk were also tested in the testing set (Table S3).

**Table 2:** Association of sporadic Alzheimer's disease risk with candidate SNPs reported in European descent among Chinese subjects in the discovery set

Chr	SNP	Position	Gene	Reported risk allele^a^	Allele frequency		Association test^b^		Association test^c^	
					SAD	Controls	OR (95% CI)	*P* value	OR (95% CI)	*P* value
1	rs6656401	207,692,049	CR1	A	0.028	0.021	1.11(0.51-2.39)	0.800	1.13(0.51-2.52)	0.769
1	rs3818361	207,784,968	CR1	A	0.354	0.378	0.93(0.73-1.19)	0.561	0.92(0.72-1.18)	0.524
2	rs7561528	127,889,637	BIN1	A	0.123	0.151	0.82(0.58-1.16)	0.258	0.87(0.61-1.23)	0.426
2	rs744373	127,894,615	BIN1	G	0.366	0.376	0.97(0.76-1.24)	0.817	0.98(0.76-1.25)	0.848
2	rs35349669	234,068,476	INPP5D	T	0.013	0.011	1.18(0.39-3.52)	0.770	1.17(0.37-3.67)	0.793
5	rs190982	88,223,420	MEF2C	A	0.864	0.849	1.18(0.84-1.68)	0.342	1.27(0.89-1.81)	0.196
6	rs9271192	32,578,530	HLA-DRB5-HLA-DRB1	C	0.144	0.112	1.30(0.91-1.85)	0.144	1.35(0.94-1.94)	0.108
6	rs9349407	47,453,378	CD2AP	C	0.202	0.118	1.95(1.39-2.74)	1.23 × 10^−4^	2.03(1.43-2.88)	8.38 × 10^−5^
6	rs11754661	151,207,078	MTHFD1L	A	0.033	0.027	1.19(0.58-2.41)	0.634	1.19(0.57-2.51)	0.647
7	rs2718058	37,841,534	NME8	A	0.754	0.797	0.79(0.60-1.05)	0.103	0.80(0.60-1.07)	0.128
7	rs1476679	100,004,446	ZCWPW1	T	0.708	0.705	1.07(0.83-1.39)	0.589	1.04(0.80-1.35)	0.786
7	rs11767557	143,109,139	EPHA1	T	0.835	0.873	0.74(0.53-1.03)	0.072	0.76(0.54-1.06)	0.109
7	rs11771145	143,110,762	EPHA1	G	0.507	0.463	1.12(0.88-1.42)	0.357	1.03(0.81-1.32)	0.803
8	rs28834970	27,195,121	PTK2B	C	0.276	0.302	0.88(0.67-1.14)	0.332	0.88(0.67-1.16)	0.367
8	rs11136000	27,464,519	CLU	C	0.832	0.806	1.18(0.87-1.60)	0.284	1.17(0.86-1.60)	0.319
8	rs569214	27,487,790	CLU	G	0.489	0.505	0.97(0.76-1.24)	0.803	0.98(0.76-1.27)	0.895
11	rs983392	59,923,508	MS4A6A	A	0.959	0.972	0.77(0.41-1.43)	0.404	0.68(0.36-1.27)	0.225
11	rs610932	59,939,307	MS4A6A	G	0.667	0.646	1.11(0.87-1.43)	0.398	1.09(0.84-1.42)	0.496
11	rs4938933	60,034,429	MS4A4A	T	0.754	0.726	1.15(0.88-1.51)	0.305	1.15(0.86-1.52)	0.345
11	rs2373115	78,091,150	GAB2	C	0.580	0.594	0.95(0.75-1.20)	0.648	0.93(0.73-1.19)	0.581
11	rs17817600	85,677,471	PICALM	G	0.037	0.043	0.85(0.45-1.59)	0.607	0.67(0.35-1.30)	0.238
11	rs3851179	85,868,640	PICALM	C	0.635	0.598	1.17(0.92-1.49)	0.194	1.19(0.93-1.52)	0.177
11	rs11218343	121,435,587	SORL1	T	0.750	0.669	1.60(1.22-2.11)	8.32 × 10^−4^	1.57(1.18-2.09)	0.002
14	rs17125944	53,400,629	FERMT2	C	0.263	0.218	1.32(1.00-1.75)	0.048	1.35(1.02-1.81)	0.040
14	rs10498633	92,926,952	SLC24A4	G	0.884	0.884	1.04(0.72-1.50)	0.838	1.10(0.76-1.61)	0.606
19	rs3764650	1,046,520	ABCA7	G	0.321	0.276	1.25(0.96-1.61)	0.097	1.26(0.96-1.64)	0.093
19	rs6859	45,382,034	PVRL2	A	0.403	0.307	1.51(1.18-1.94)	0.001	1.22(0.93-1.59)	0.149
19	rs157580	45,395,266	TOMM40	A	0.531	0.473	1.29(1.02-1.65)	0.036	1.04(0.80-1.35)	0.769
19	rs2075650	45,395,619	TOMM40	G	0.236	0.084	3.19(2.25-4.52)	7.87 × 10^−11^	2.50(1.60-3.91)	6.16 × 10^−5^
19	rs3865444	51,727,962	CD33	C	0.838	0.840	0.97(0.70-1.36)	0.878	1.03(0.73-1.45)	0.888

Abbreviation: SAD, sporadic Alzheimer's disease; Chr, chromosome; SNP, single nucleotide polymorphism; OR, odds ratio; CI, confidence interval.

a Risk allele reported in European population.

b Association test was adjusted for sex, age (age at onset for SAD patients and age at examination for control subjects).

c Association test was adjusted for sex, age (age at onset for SAD patients and age at examination for control subjects) and APOE ε4 status (0 or 1).

### GRS calculation and its discriminative performance analysis in the testing set

To assess the cumulative effect of multiple SAD risk-associated SNPs in predicting SAD risk, we calculated GRS for subjects in the independent testing set based on all 6 implicated SNPs in the discovery set (Table 3). The median GRS was significantly higher in SAD cases than in controls, *P* < 0.001. AUC of GRS in discriminating SAD cases from controls was 0.63, higher than that of *APOE* (0.60).

**Figure 1:**
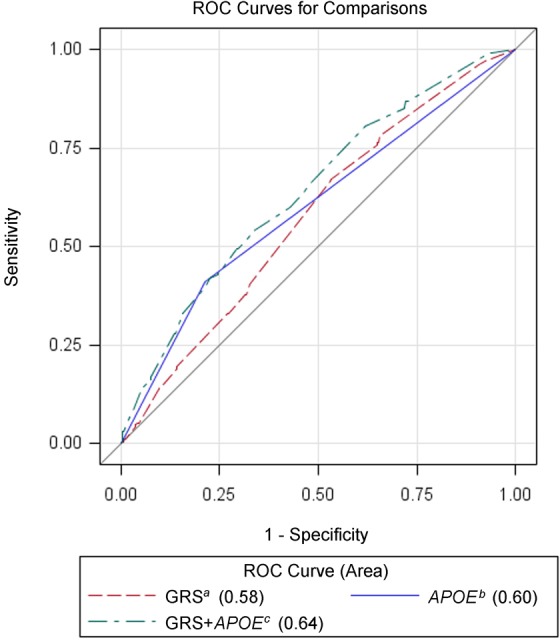
Receiver operating characteristic curves for genetic models among Chinese subjects in the testing set ^a^ Modified genetic risk score (GRS) based on 3 SNPs (rs9349407, rs11218343 and rs17125944). ^b^ *APOE ε4* status (0 or 1). ^c^ Combination of modified GRS and *APOE*.

Considering that the effect of GRS may be confounded by *APOE*, we calculated a modified GRS by removing three SNPs that are related to *APOE*: two SNPs that were no longer significantly associated with SAD risk after adjusting for *APOE* genotypes (rs6859 and rs157580) and one SNP that was in strong LD with *APOE* genotypes (rs2075650). In the independent testing set, the modified GRS was significantly higher in SAD cases than in controls, *P* = 0.002 (Table 3). The AUC of the modified GRS was 0.58. When this modified GRS was combined with *APOE* genotypes, the AUC was 0.64, significantly higher than that of *APOE* alone (0.60), *P* = 0.003 (Figure 1).

### Association analysis of GRS and SAD risk

When the modified GRS was analyzed in subjects stratified by the *APOE ε4* status in the testing set, similar trends were observed in both *APOE ε4* carriers and non-carriers, although the association and discriminative performance of modified GRS was slightly stronger in non-carriers (Table 3).

**Table 3:** Association of genetic risk score and SAD risk in the testing set

	# of subjects (SAD/Controls)	Mean GRS		Median GRS			
Sample set		SAD	Controls	SAD	Controls	*P* value	AUC
GRS based on 6 SNPs	227/378	2.48	1.15	0.85	0.55	<0.001	0.63
APOE ε4 carriers	93/81	4.57	2.47	2.51	1.69	0.003	0.64
APOE ε4 non-carriers	134/297	1.03	0.79	0.53	0.47	0.101	0.55
							
Modified GRS based on 3 non APOE-related SNPs	227/378	1.02	0.91	0.87	0.87	0.002	0.58
APOE ε4 carriers	93/81	1.01	0.92	0.87	0.87	0.187	0.56
APOE ε4 non-carriers	134/297	1.02	0.91	0.87	0.87	0.006	0.58

Abbreviation: SAD, sporadic Alzheimer's disease; GRS, genetic risk score

## DISCUSSION

The primary purpose of this study was to assess performance of multiple risk-associated SNPs for predicting SAD risk in the Han Chinese population. To achieve this goal, we firstly identified SNPs that were associated with SAD risk among Chinese in a discovery set. We then assessed the cumulative effect of these implicated SNPs, as measured by GRS, on association of SAD risk and ability to discriminate SAD patients from non-dementia controls in the testing set. Furthermore, to assess whether the predictive performance of multiple SAD risk-associated SNPs are independent of *APOE* genotypes, we calculated a modified GRS that based on three SAD risk-associated SNPs that are independent of *APOE* genotypes. With this rigorously strategy, we demonstrated that modified GRS was able to discriminate SAD patients from controls, with an AUC of 0.58. When combined the modified GRS with *APOE*, the AUC increased to 0.64, significantly higher than *APOE* alone (0.60), *P* = 0.003.

To our knowledge, this is the first report assessing cumulative effect of multiple AD risk-associated SNPs on association and discrimination of SAD. It is well recognized that effect of individual SNPs on AD risk is modest. However, it is hypothesized that cumulatively they have a stronger effect. As demonstrated in this study, the AUC of modified GRS based on three implicated SAD risk-associated SNPs (0.58) was similar to that of well-established *APOE* (0.60). This result offers empirical evidence to support this cumulative effect hypothesis and provides basis for additional larger and more comprehensive studies to further test the hypothesis in Chinese and European descent. With more established AD risk-associated SNPs (such as in European descent), it is expected that cumulative effect will be stronger.

The findings that SAD risk-associated SNPs are associated with SAD risk in both *APOE ε4* allele carriers and non-carriers and that they add value to *APOE* in discriminating SAD cases from controls are important. On one hand, these results suggest SAD risk-associated SNPs play similar roles in the etiology of SAD among *APOE ε4* allele carriers and non-carriers. On the other hand, it is practically important in assessment of SAD risk. The *APOE ε4* allele has been found to be the strongest risk factor for SAD [9-12]. A meta-analysis of clinical and autopsy-based studies demonstrated that individuals with *ε4* allele have increased AD risk compared with ε3/ε3 genotype in Caucasian population (OR was 2.6, 3.2, and 14.9 for individuals with *ε2*/*ε4*, *ε3*/*ε4*, and *ε4*/*ε4*, respectively) [32]. However, the AUC of *APOE* alone in discriminating SAD remains moderate (for example, AUC was 0.62 and 0.60 in the discovery set and testing set of our study, respectively). Furthermore, it is recognized that 40~50% of SAD patients do not carry *APOE ε4* allele [11, 13]. This number was even higher in our study where 58.6% SAD patients did not carry *APOE ε4* allele (Table 1). Therefore, identifying tool for better risk assessment of AD risk, especially among subjects without *APOE ε4* allele is necessary. Better risk assessment may identify subjects at higher risk for SAD for targeted prevention. Subjects with a higher risk for SAD may be more motivated to take action to prevent AD through reducing life-style risk factors associated with AD.

Family history is another well-established risk factor for AD. The relative risk of AD for those with at least one first degree relative with dementia was estimated at 3.2-3.8 [33-35]. Unfortunately, we could not assess the effect of family history on AD risk in this study because we focused on SAD in this study, and none of the AD patients had a positive family history by our inclusion criteria. However, the fact that we demonstrated GRS based multiple inherited risk-associated SNPs is a predictor of SAD risk among patients without a known family history indirectly suggests family history alone is not sufficient to capture inherited risk for AD. In contrast to GRS that is based on individual's own risk-associated SNPs, family history is an indirect measurement of familial risk (inherited and shared household environment) through their relatives, and is therefore influenced by the number, age, and competing mortality of their relatives. This limitation is more prominent for late age onset diseases such as AD. Thus, lack of a known family at the time of examination may not necessarily indicate that individuals are at lower risk for AD. Studies are needed to assess the combined performance of family history, GRS, and *APOE* in assessing AD risk.

We randomly divided our study subjects into two sets of equal sample size: discovery and testing. The major advantage of this approach is that we can identify SAD risk-associated SNPs in Han Chinese and obtain their OR in the discovery set and then objectively assess the performance of these SNPs in the independent testing set. However, this approach reduced the statistical power to detect association of SNPs with SAD risk. Among the 30 SNPs tested in the discovery set, 19 SNPs had the same direction of association as in the studies of European descent, although only 6 SNPs reached the statistical significance of *P* < 0.05. Larger sample size may be needed to confirm additional AD risk-associated SNPs in Han Chinese.

Several case-control studies on association of AD risk-associated SNPs reported in GWAS of European descent with AD risk in Chinese population were published in the last several years. However, few SNPs were consistently implicated among these studies. For example, Chen et al. [21] evaluated 7 SNPs (rs3818361 and rs6656401 in *CR1*; rs11136000, rs2279590, and rs9331888 in *CLU*; rs3851179 and rs541458 in *PICALM*) among 462 AD patients and 350 control subjects from southern Chinese population. Of the 7 SNPs, rs6656401 (*P* = 0.035) and rs3818361 (*P* = 0.029) in *CR1*, and rs11136000 in *CLU* (*P* = 0.038) were confirmed; rs3851179 in *PACALM* showed significant association with LOAD only in *APOE ε4* non-carriers (*P* = 0.028). Tan et al. [25] assessed a total of 10 SNPs among 612 sporadic late-onset AD (LOAD) patients and 612 control subjects from northern Han Chinese, including 2 in *BIN1* (rs7561528 and rs744373), 2 in *ABCA7* (rs3752246 and rs3764650), 3 in the *MS4A* gene cluster (rs4938933, rs610932, and rs670139), and 1 each in *CD2AP* (rs9349407), *CD33* (rs3865444), and *EPHA1* (rs11767557). Based on a multivariate analysis, rs610932 in *MS4A6A* (*P* = 0.019) and rs3865444 in *CD33* (*P* = 0.017) were confirmed; rs7561528 in *BIN1* was confirmed only in *APOE ε4* carriers (*P* = 0.039). Ma et al. [26] conducted a replication study of rs11754661 and rs2073067 in *MTHFD1L* among 582 LOAD subjects and 607 healthy controls from northern Han Chinese. The rs11754661 was confirmed (*P* = 0.016). Liu et al. [23] evaluated and confirmed the association of rs3764650 in *ABCA7* with SAD in 350 SAD and 283 non-demented elderly controls from Han Chinese, *P* = 0.004. Ma et al. [27] investigated the association of three SNP (rs157580, rs2075650 and rs11556505 in *TOMM40*) with LOAD among 787 LOAD patients and 791 healthy subjects. The rs157580 (*P* < 0.001) and rs2075650 (*P* = 0.001) were confirmed. Among these confirmed SNPs (rs3818361, rs6656401, rs7561528, rs11754661, rs11136000, rs610932, rs3851179, rs3764650, rs3865444, rs157580 and rs2075650), 2 SNPs (rs157580 and rs2075650 in *TOMM40*) were also found to be significantly associated with SAD in our study. Except for 2 SNPs (rs3818361 and rs7561528), the rest SNPs have the same direction of association with previous findings in Chinese. Multiple factors may contribute to these different confirmed SNPs, including small sample size, different criteria for AD patients and controls, and different genetic background between northern and southern Han Chinese.

The three SNPs, independent of *APOE* genotypes and used in GRS calculating to predict SAD risk in the study, are rs9349407 in *CD2AP* (intron 1), rs11218343 in *SORL1* (intron 21), and rs17125944 in *FERMT2* (intron 14). The functions of these 3 genes have been reported to be relevant to the development of AD. Both *CD2AP* and *FERMT2* have been implicated in cell adhesion [36, 37]. In Drosophila model of AD, *CD2AP* and *FERMT2* were identified as modifier of Tau neurotoxicity, which related to neurofibrillary tangle pathology in AD [38]. *SORL1* encodes a neuronal sorting protein that binds APP protein and directs it towards the endosome-recycling pathways [39] and variants in *SORL1* were significantly associated with cerebrospinal Aβ42 levels [40], which reflect the metabolic process in brain and was used to aid the diagnosis of AD at an early stage of disease.

In summary, results from this well-designed but underpowered study provided preliminary evidence that multiple AD risk-associated SNPs can be used to supplement *APOE* to better define individual's risk for AD. Larger studies are justified to formally test the hypothesis and assess its predictive performance.

## MATERIALS AND METHODS

### Study population

Subjects included in this study (515 SAD patients and 770 cognitively normal controls) were Chinese Han, and were recruited during 2008-2013. SAD patients, comprising early- and late-onset SAD (age at onset ranged from 45 to 88 years), were recruited from Huashan Hospital in Shanghai and were diagnosed as probable AD according to DSM-IV-R and NINCDS-ADRDA criteria [41, 42]. All SAD patients reported no family history of AD. The cognitively normal controls were recruited from communities in Shanghai and were carefully evaluated based on mini-mental state examination (MMSE) and years of education. They were frequency matched for SAD cases by gender and age. Two senior neurologists reviewed all data and confirmed the diagnosis. *APOE* genotype status, measured by method described by Donohoe et al. [43], was available in cases and controls. This study was approved by the ethics committee of Huashan Hospital and written informed consents were completed for all study subjects.

After genotyping, subjects with a missing rate of > 20% were removed from the study (56 SAD patients and 19 control subjects). The retained subjects (459 SAD patients and 751 controls) were randomly divided into discovery set (232 cases and 373 controls) and testing set (227 cases and 378 controls). Association of AD risk-associated SNPs reported in European descent with SAD risk was firstly tested in the discovery set. Significant SNPs were used to calculate GRS in the testing set.

### SNP selection

A total of 33 SAD risk-associated SNPs were selected using the following criteria: 1) association with AD risk in European population exceeded the threshold of a genome-wide significance level (*P* < 5 × 10^−8^) and published before Jan 2014; 2) if multiple SNPs that are in strong linkage disequilibrium (LD) met the above criterion, defined by pairwise r^2^ > = 0.2 estimated from the HapMap CHB (Han Chinese in Beijing, China) population, the most commonly cited SNP was selected. The information of all SNPs that be chosen was listed in Table S4.

### SNP genotyping and Quality control

Genotyping of selected SNPs was performed using the Sequenom MassArray system (iPLEX; Sequenom, Inc. San Diego, CA) at the Centre for Genomic Translational Medicine and Prevention, School of Public Health, Fudan University. Duplicates from two subjects and two water samples (negative control) were included in each 96-well plate for genotyping quality control. All assays were conducted blinded to case-control status. The overall concordance rate was 100% among the duplicated quality control samples. A quality control was conducted to select the samples (mentioned in “*Study Population”* section) and SNPs for further analysis. SNPs with a missing rate of > 5%, the minor allele frequency (MAF) of < 0.01 in either cases or controls, or with Hardy-Weinberg equilibrium (HWE) test at *P* < 0.001 among controls were excluded.

### Statistical analysis

Differences between cases and controls were tested using t-test for quantitative variables and Chi-square test for qualitative variables. Associations between SNPs and SAD risk were tested for each SNP using an additive model adjusted for 1) sex, age (age at onset for SAD patients and age at examination for control subjects); 2) sex, age (age at onset for SAD patients and age at examination for control subjects) and *APOE ε4* status (0 or 1). The allelic odds ratio (OR) and 95% confidence intervals (CI) were estimated using a logistic regression model.

GRS for each subject was calculated based on SAD risk-associated SNPs established in the discovery set using the method described by Pharoah et al. [44]. Briefly, 1) the allelic OR of each SNP was obtained from the discovery set, 2) the genotypic OR of each SNP was estimated from the allelic OR assuming a multiplicative model, 3) the risk relative to the average risk in the population was calculated for each genotype based on genotypic OR and genotype frequency in the HapMap CHB (Han Chinese in Beijing, China) population, and 4) a GRS was obtained by multiplying the risks relative to the population of all SNPs. Therefore, a GRS of 1.0 indicates an average risk in the general population.

Non-parametric analysis (Wilcoxon Rank Sum Test) was used to test association of GRS and SAD risk. The performance of GRS in discriminating SAD cases from controls was evaluated using the area under the receiver operating characteristic curve (AUC). Difference in AUC between two predictive model was tested using the method described by DeLong and colleagues [45].

All statistical analyses were performed using the PLINK software (version 1.07) [46] and SAS software (version 9.2; SAS Institute, Cary, NC). All statistical tests were two-sided.
